# Advancing synthetic biology through cell-free protein synthesis

**DOI:** 10.1016/j.csbj.2023.05.003

**Published:** 2023-05-04

**Authors:** Ke Yue, Junyu Chen, Yingqiu Li, Lei Kai

**Affiliations:** a School of Life Sciences, Jiangsu Normal University, Xuzhou 22116, China

**Keywords:** Cell-free protein synthesis, Synthetic biology, Minimal cell, Metabolic engineering, Biomanufacturing, In vitro diagnostics

## Abstract

The rapid development of synthetic biology has enabled the production of compounds with revolutionary improvements in biotechnology. DNA manipulation tools have expedited the engineering of cellular systems for this purpose. Nonetheless, the inherent constraints of cellular systems persist, imposing an upper limit on mass and energy conversion efficiencies. Cell-free protein synthesis (CFPS) has demonstrated its potential to overcome these inherent constraints and has been instrumental in the further advancement of synthetic biology. Via the removal of the cell membranes and redundant parts of cells, CFPS has provided flexibility in directly dissecting and manipulating the Central Dogma with rapid feedback. This mini-review summarizes recent achievements of the CFPS technique and its application to a wide range of synthetic biology projects, such as minimal cell assembly, metabolic engineering, and recombinant protein production for therapeutics, as well as biosensor development for in vitro diagnostics. In addition, current challenges and future perspectives in developing a generalized cell-free synthetic biology are outlined.

## Graphical Abstract

**Figure:**
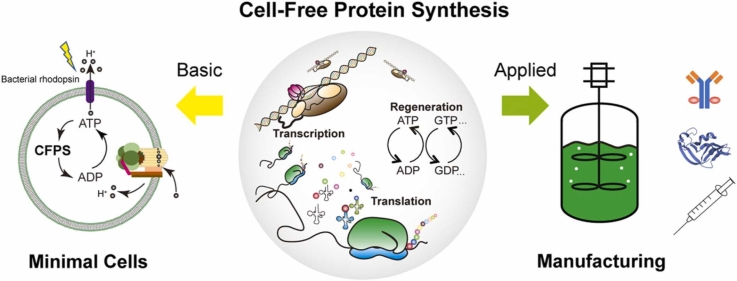

## 1 Introduction

The call to move towards a more sustainable, green industry has been a catalyst for the growth of biotechnology, shifting our economy away from industries dependent on petroleum and toward bio-based industries. Synthetic biology is an interdisciplinary field that combines biology, engineering, and technology to create products and services through living systems. Its rapid growth has the potential to revolutionize biotechnology, medicine, and the environment [1], [2], [3]. Recent breakthroughs in the genomic editing tools of the CRISPR/Cas systems [4], [5], [6], in conjunction with the earlier molecular tools of synthetic biology, have allowed the precise and efficient engineering of biological systems to generate a wide range of products, including renewable energy sources, crop varieties, food and medical products, and other environmentally friendly products. Although the ongoing development of such gene modification techniques has provided powerful programming tools for cell factories, there are still inherent constraints [7]. The coupling of the maintenance of the living system and the synthesis of desired products defines the upper-limit efficiency of the whole synthetic system. In addition, the complex cellular system and chassis behavior of different model host cells result in inconsistency from case to case.

With its distinct advantages for applications in synthetic biology, the Cell-free protein synthesis (CFPS) approach is emerging to solve the aforementioned general constraints of cells [8], [9], [10], [11]. The CFPS system employs a minimum enzymatic apparatus for transcription, translation, and energy regeneration, derived either from cell extracts or pure enzymes [12], [13]. Born as a simple and streamlined reconstituted system, CFPS naturally overcomes this inherent limitation of a living cell and provides direct access to its essential activities. Such an open nature of the CFPS enables the first-ever programming of modular cellular mimicking processes with active transcription and translation support. In addition, non-native chemicals can be introduced directly into the system, allowing greater flexibility in the selection of regulating reagents [14], [15].

**Fig. 1:**
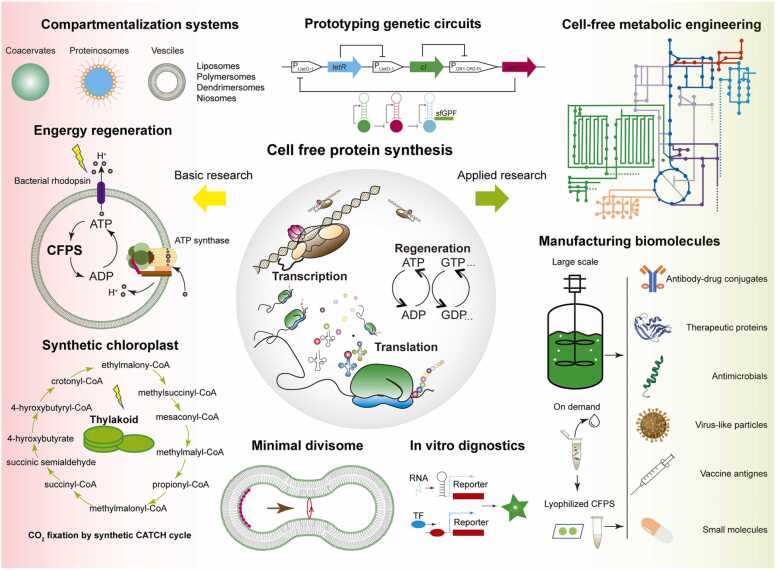
Applications of cell-free protein synthesis in both fundamental and applied research in synthetic biology. With reconstituted transcription and translation machinery, the CFPS system (adapted from Ref. [25] with permission from Copyright 2017, Elsevier B.V.) is extensively utilized in the construction of minimal cellular mimicry systems within different compartmentalization systems (adapted from Ref. [7] with permission from Copyright 2019, WILEY-VCH), such as energy regeneration, metabolism (photosynthesis via the synthetic metabolic pathway: the crotonyl-CoA/ethylmalonyl-CoA/hydroxybutyryl-CoA (CATCH) cycle), and the reconstitution of minimal divisome. CFPS synthesis has been accepted as a basic method for prototyping genetic circuits (adapted from Ref. [109] under Creative Commons CC BY license 2019) metabolic engineering, and large- and small-scale biomanufacturing in the applied sector. The lyophilization of CFPS offers a long shelf life and non-cold chain shipping, allowing on-demand production of antibody-drug conjugates, therapeutic proteins, antimicrobial peptides, virus-like particles, vaccine antigens, and small-molecule medicines. Furthermore, the coupling of CFPS with other biosensors offers considerable diagnostic potential *in vitro*.

The application of the CFPS technique came much later to the field of synthetic biology than its application as tools for discovering and illustrating basic principles of biological systems. The first well-known application of the CFPS technique was done by Nirenberg and Matthaei [16], [17], [18] to decipher the genetic codes. Later, with continuous improvement in efficiency, it gradually developed as a complementary tool for the production of recombinant proteins, in particular, for membrane proteins and toxins that are not well expressed in cells [15], [19], [20], [21], [22], [23]. In addition to protein production, the application of the CFPS technique to the broad field of synthetic biology probably came first to the field of synthetic cell construction via a bottom-up strategy [24], [25], [26], [27]. This distinct field seeks to obtain a plausible route to the origin of life from the bottom up, which starts with simple molecules, such as fatty acids, DNA or RNA molecules, to establish cell mimicry systems [28], [29]. Such a system can possess certain essential characteristics of a living cell or carry out certain basic activities [30], [31]. However, when the CFPS technique is applied, more complex modular systems can be established beyond simple enzyme-catalyzed reactions, such as energy generation, metabolism, template-guided self-replication, growth, and division, which can be further integrated with additional regulatory machinery (Fig. 1) [32], [33]. In addition, CFPS also shows great potential to revolutionize cell-based synthetic biology with product-driven applications [33], [34]. The fast cycle of CFPS has greatly shortened the period of the typical design-build-test (DBT) circle, in particular for tasks such as metabolic engineering [35], protein-directed evolution [36], [37], [38], [39], and *de novo* protein design [40]. Last but not least, the combination of the CFPS technique and DNA manipulation tools, such as DNA amplification and editing, further extends its application in biomedical applications of in vitro diagnostic and portable analytical tools (Fig. 1, Table 1).

**Table 1:** Summary of the major applications of CFPS in synthetic biology.

Applications	Specifications	References
Construction of minimal cellular mimicry systems	Compartmentalization	Liposomes [111], polymersomes [113], proteinosomes [120], [121], dentrimersomes [122], [123], [124], encapsulins [125], [126] and niosomes [127], [128] were developed for compartmentalization
	Energy regeneration	ATP regeneration via a light-driven proton pump and an ATP synthase [138], [139]
	Metabolism	Semi-synthetic chloroplast [140]
	Self-replication	Replication via phi29 polymerase and PURE system [141,142]
	Growth and Division	*De novo* synthesis phospholipids [144]; reconstitution of proto-ring within a GUV [155]
	Communication, motility and evolution	See references [156], [157], [158]
Products-oriented applications	Metabolic engineering	Achieve 1.5 g/L production of n-butanol [165], 17.6 g/L of mevalonate [164]
	Manufacturing biomolecules	Antibody-drug conjugates, therapeutic proteins, antimicrobials, virus-like particles, vaccine antigens, small molecules [168], [169]
	*In vitro* diagnostics	Develop a portable device for the detection of anhydrotetracycline, Ebola virus RNA, and various small molecules [183], [184]

## 2 Constant evolution of CFPS systems

Since its inception in the 1960 s, the CFPS system has undergone extensive systematic optimization to increase its efficiency. This optimization has been extended to all major components, including extract [41], [42], [43], [44], [45], [46], template [47], [48], [49], energy source [50], [51], [52], [53], buffer [53], [54], [55], [56], [57], and incubation and reaction settings [41], [58]. Productivity continues to increase from around 0.1 mg/mL to above 4.0 mg/mL in batch configurations for crude lysate-based CFPS [59]. In terms of the fully reconstituted CFPS system, protein synthesis using recombinant elements (PURE) has also been systematically optimized, resulting in an improvement in efficiency from micrograms to sub-milligrams per mL in a batch configuration [60]. The pioneering work by Spirin and co-workers greatly improved the efficiency of the resulting CFPS system by a gradient-driven passive compound exchange through a dialysis membrane, which provided not only a large pool of substrate precursors, but also a sink for removal of inhibitory by products such as free phosphate. Besides in solution, the CFPS reaction can also be lyophilized as pellets or on porous matrices such as filter paper or cellulose matrices, which allowed the storage under room temperature and could be activated later via rehydration (see the section ‘*Manufacturing Biomolecules’ and* ‘*New Trends in In Vitro Diagnostics*’) [61], [62], [63]. The detailed development of various CFPS was reviewed in a set of review papers by us and other groups [22], [25], [64], [65], [66].

In addition to the PURE system, CFPS relies on crude cell lysate to provide the enzymatic pool to support gene expression [67]. Currently, the sources of the extract are extremely diverse, including prokaryotic, eukaryotic, and archaeal cells, from model host cells such as *E. coli* [41], [42], *Bacillus subtilis* [68], yeast [69], insect cells [70], [71], rabbit reticulocyte [72], wheat germ [73], [74], [75], tobacco cell [76], rice callus cells [77], CHO cells [78], [79], Hela [80], to non-model organisms such as *Thermococcus kodakaraensis* [81], *Klebsiella pneumoniae* [82], *Vibrio natriegens* [83], *Clostridium thermocellum* [84] and Streptomyces [85], which were reviewed with regard to their advantages and disadvantages by Kubick et al. [86]. In conclusion, prokaryotic systems have the advantage of high productivities but no inherited post-translational modifications, whereas eukaryotic systems have the advantage of inherited post-translational modifications but low total expression yields. Furthermore, the limited modification machinery from eukaryotic cell extract may result in the heterogeneity of the proteins that are produced. Due to the accessibility of most molecular tools, *E. coli*, the most thoroughly researched model cell, remained the predominant source for synthetic biology. Furthermore, recent studies have shown that some post-translational modifications, such as phosphorylation [87], [88], glycosylation [89], and lipidation [90], could be performed successfully with *E. coli*-based CFPS systems by incorporating functional extracts or pure enzymes containing the associated modification machinery. Therefore, we focus on the *E. coli-*based CFPS system in the following parts.

Within a cell, gene expression is a carefully regulated process that ensures the spatial-temporal distribution of hundreds of proteins to perform their required activities. The evolution of genetic circuits is focused on the regulation of gene expression networks, which have been studied intensively in vivo using numerous approaches [91]. With inheritance from the crude cell lysate, the CFPS system has the ability to activate in vivo validated genetic circuits. However, relatively fewer incidents have been reported compared to in vivo. For example, experiments to control CFPS transcription rates were conducted using different polymerases, such as T7 and bacterial *E. coli* RNA polymerases [92], [93]. Additionally, different T7 promoters were used to regulate transcription and translation rates in CFPS [94]. To further engineer cell-free synthetic gene circuits, a cell-free expression toolbox was designed with 13 different *E. coli* sigma factors, two bacteriophage RNA polymerases, and a set of repressors. This toolbox allowed the design and testing of various circuit motifs, such as multiple-stage cascades, an AND gate, negative and positive feedback loops, transcriptional cascades with a protein-regulated incoherent feedforward loop, and in vitro ring oscillators [95]. In addition to proteins, RNA molecules can also regulate transcription, translation, and catalysis and provide an attractive alternative to regulatory elements [96], [97], [98], [99], [100], [101]. Riboswitches, for example, are located in the non-translated region of mRNA and can up- or down-regulate gene expression in response to ligand binding [102], [103], [104], [105], [106], [107]. Synthetic theophylline-responsive riboswitch and natural adenine-sensing *B. subtilis* riboswitch have been established for robust in vitro on-off switching in water-in-oil emulsions and vesicles [108].

## 3 Application of CFPS to the construction of minimal cells

### 3.1 Compartmentalization

The prerequisite to distinguish a living cell from the non-living environment is a physical boundary that offers the basic control of mass exchange between the internal compartment and the outer space. Anything that stabilizes phase separation can be used as a material to form synthetic compartments [110]. Inspired by nature, extracted or synthetic phospholipids are often used for their ability to form cell-like versicles by self-assembly into a bilayer [111]. Similar to the structure of a phospholipid molecule, synthetic polymers, such as block copolymers [112], can also self-assemble into vesicles [113] and can be tuned to control the basic properties of the formed vesicles, such as size, membrane thickness [114], rigidity and permeability [115], surface properties [116], and selectivity of the encapsulated materials [117]. Hybrid vesicles that form homogeneous or phase-separated membranes have been reported to depend on the specific application requirement [118], [119]. In addition, alternative compartmentalization systems such as proteinosomes [120], [121], dentrimersomes [122], [123], [124], encapsulins [125], [126], and niosomes [127], [128] were reported. Following the basic principles for forming a self-assembled compartment, they offer superior properties, such as stability, encapsulation efficiency, and biocompatibility with natural cells. Moreover, other phase-forming materials, such as hydrogel [129], [130] and coacervate [131], [132], by liquid-liquid phase separation, can also be used as membraneless compartmentalization methods, mimicking the membrane organelles in cells [133], [134], [135]. However, in addition to the physical boundary, the cell membrane functions as the matrices and catalytic interfaces for many processes like signaling, which certainly require addition factors like membrane proteins, as discussed in the ‘Summary and Perspective’ section.

### 3.2 Integration of individual synthetic modules

Before the application of the CFPS technique in the construction of minimal cells, individual modules such as energy regeneration, self-replication, growth and division, metabolism, motility, and communication could be carried out by a few simple molecules. However, the success of encapsulating the CFPS system in a synthetic microcompartment would already be a multifunctional synthetic cell that at least contains modules for energy regeneration, transcription, and translation machinery. In 2004 Noireaux et al. successfully encapsulated CFPS within phospholipid vesicles by emulsion transfer, which could produce 30 µM protein in 4 days [136]. Furthermore, expression could be tuned by introducing regulatory molecules, such as transcription factors (see the above section on genetic circuits). The CFPS system contains a number of molecules to fuel the reaction, which are normally the energy precursor within glycolysis pathways [53], or high-energy phosphate compounds such as acetyl phosphate [137], creatine phosphate [43]. Besides the energy precursors used in the CFPS system, the light-induced proton gradient was adopted to fuel gene expression in the CFPS system through a light-activated proton pump (bacteria rhodopsin) and an ATP synthase within the membrane of the synthetic cell [138], [139]. In addition to energy regeneration, the cell uses metabolism to obtain building blocks required for self-reproduction. The beauty of the cell metabolism network is that the entire metabolism network is auto-regulated to maintain homeostatic control while providing continuous materials and energy. Recently, a show-off case was reported in which light-driven CO_2_ fixation could be carried out by a synthetic chloroplast consisting of natural and synthetic parts [140]. As the ultimate goal of building a living cell, self-reproduction allows the generation of offspring that have the same genetic materials, which is the critical step for continuous existence and evolution. In order to be able to self-reproduce, a cell would need several essential characters including: 1) self-replication of information molecule (genetic material); 2) growth and division. Therefore, these functions are the first goal to be achieved toward a minimal cell. In one case, the Danelon group showed [141] a self-replicated artificial cell that was able to self-replicate by co-encapsulating the DNA template encoded with the phi29 polymerase via the PURE system. In this case, the DNA of phi29 could be replicated when the coding proteins were expressed by the PURE system. In another case, Libicher et al. [142] demonstrated a replication of the 116 kb multipartite genome (distributed in 11 plasmids) via the PURE system. The size of 116 kb already matched the minimum genome. However, further efforts are still needed to check if this could be achieved in any synthetic microcompartments. Being able to self-replicate, cells need to be able to grow and divide to reproduce in a sustainable way. As the first physical boundary of a cell, the materials that hold the compartment define the volume of the system and need to be reproduced. Several examples have shown the growth of different microcompartments without enzymes [143]; however, we envision that *de novo* synthesis of lipid molecules is vital for autonomous self-reproduction. As the critical step, recent studies showed the successful synthesis of PE and PG within the synthetic cell by activating the synthesis of correlated enzymes by the encapsulated CFPS system [144]. Finally, a division step would complete the self-reproduction process. Although division could be achieved through many mechanical processes by introducing appropriate shear forces [145], self-autonomous division would require several key steps to ensure a symmetric distribution of materials in the resulting daughter cells [146]. In bacterial cells, such as *E. coli*, binary division is initiated by forming a proto-ring, which ultimately leads to complete division by invagination [147], [148], [149], [150]. Pioneering work by the Schwille group has shown that positioning the proto-ring at the middle of the cell only required three self-organized proteins-Min C, D, and E from *E. coli* [151], [152], [153], [154]. Furthermore, one of their recent studies has shown a successful positioning of the proto-ring with Min C, D, E, FtsA, and FtsZ by *de novo* synthesis via a PURE system within a GUV [155]. More interestingly, a ring-induced shape transformation of the formed GUVs was observed, which might be the leading forces to trigger further deformation until the GUVs were divided. Many other synthetic systems have been successfully established that could enable communication [156], mobility [157], and evolution [158] with the integration of the CFPS technique. Therefore, we would envision a more mature multifunctional minimal cell, merging with the continuous efforts in the near future.

## 4 Application of CFPS to products-oriented synthetic biology

### 4.1 Cell-free metabolic engineering (CFME)

Unlike cell metabolic engineering, cell-free metabolic engineering involves the engineering of metabolic pathways in a cell-free environment through purified enzymes or crude cell lysates [159]. The major advantage of CFME is that it is much faster and more efficient, since it eliminates the need for iteration and selection steps when producing a desired product. CFME also avoids problems associated with cell-based approaches, such as cell toxicity and genetic instability. In addition, it is much easier to optimize and troubleshoot in CFME, as the reactions occur in vitro, allowing more flexibility. Finally, CFME enables the development of more complex metabolic pathways and the production of more diverse products. Long before the introduction of CFPS, enormous efforts have been made to synthesize valuable chemical compounds and natural products in bioreactors using enzymes [160], [161], [162]. However, it became extremely challenging and labor intensive to produce individual purified enzymes and combine them with proper stoichiometry for the best conversion efficiency. Recent cases gave us an overview of the current extremes of what could be achieved by purified enzymes. The Bowie group [163] reconstituted a cell-free monoterpene pathway with 27 purified enzymes, which achieved more than 95 % conversion yield and 15 g/L titer. Such a higher titer is more than an order of magnitude higher than the lethal concentrations for cell-based systems.

Pioneered by the group of Jewett [35], they proposed a novel CFPS-based metabolic engineering framework to build biosynthetic pathways. This process involved directly synthesizing each enzyme of a biosynthetic pathway in vitro using cell-free lysates and combining multiple crude lysates to initiate the DBT cycle. Enzyme-rich lysate performed catalytic tasks in place of separately isolated enzymes. Applying this strategy to mevalonate biosynthesis [164], the CFPS system produced 17.6 g/L (119 mM) of mevalonate in 20 h, in contrast to the initial titer of 1.6 g/L produced in 9 h. Using the same mechanism, a prototype of n-butanol biosynthesis was developed. The system produced 1.2 g/L of n-butanol by using natural glycolytic enzymes to convert glucose to acetyl-CoA and heterologous enzymes to convert acetoacetyl-CoA to n-butanol. In less than a day, the researchers studied four Ter homologs and three AdhE homologs that replaced some of the initial Ter and AdhE enzymes. They also demonstrated the use of linear DNA templates, which eliminated the need for laborious cloning procedures and resulted in an increase of up to 1.5 g/L in the synthesis of n-butanol [165]. In conclusion, CFPS-based metabolic engineering provides significantly faster DBT cycles than cell-based metabolic engineering and eliminates enzyme purification processes.

### 4.2 Manufacturing biomolecules

Proteins are major macromolecules that are essential for the structure and function of living cells, providing structure to cells, acting as enzymes to catalyze biochemical reactions, and performing or regulating a variety of other metabolic processes. Therefore, as an important recombinant protein production tool with unique advantages, the CFPS system has been applied to produce various therapeutic protein products, covering different cytokines, cytotoxins, antibodies, vaccine antigens, virus-like particles and antimicrobials, which were reviewed elsewhere [8], [166], [167]. What we want to emphasize is the flexibility of deploying the CFPS system in a variety of application environments, which suits both large-centralized industrial-scale production and small-batch production of therapeutic and laboratory reagents. Large-scale CFPS has been utilized in commercial efforts, such as Sutro Biopharma, Inc., which was able to produce up to 1000 liters of cell-free reactions [167]. This method took advantage of fast synthesis outside the cell, which greatly sped up the drug development process. Different from large centralized infrastructures, the CFPS can provide a flexible on demand protein production. As pioneered by Pardee et al., CFPS can be lyophilized to allow convenient storage and transportation conditions [65]. Lyophilized CFPS systems can be used for the decentralized, small-batch production of therapeutics and reagents, with potential uses in global health and emergency response. Furthermore, they demonstrated the production of more than 50 compounds, including vaccines, antibodies, antimicrobial peptides, and small molecules, using the same approach [168]. Recently, the Jewett group managed to provide another example of protective conjugate vaccines [169]. These products can be created outside of the laboratory setting and have the potential to revolutionize the field of bio-manufacturing.

Because of the limited amount of proteinogenic or canonical amino acids, the engineering of proteins with distinct chemical activities has been hampered. Non-canonical amino acids (NCAAs) and amino acid analogs could offer new functionalities to proteins, such as altered activity, enhanced stability, alternative post-translational modifications and more, which is particularly useful in the fields of drug discovery, bio-catalysis, and protein design when site-specific incorporation of NCAAs is possible [170]. The introduction of an NCAA at a specific position within the protein sequence requires the presence of an aminoacyl-tRNA synthetase specific for the desired NCAA, a tRNA molecule with an anticodon complementary to the NCAA's codon, and a genetically encoded amino acid specific enzyme, such as a codon-specific ribosome [171], [172], [173], [174], [175]. Several groups have done pioneering work [176]. However, difficulties persist in selecting NCAAs, which must be able to easily traverse membrane barriers. Although CPFS provided an open environment and unique advantages for selective multiplexed inclusion, the development of multidomain proteins or protein complexes would benefit more from the use of this technique [177], [178], [179].

### 4.3 New trends in in vitro diagnostics

In combination with DNA manipulation techniques, in particular the CRISPR/Cas-based system, the CFPS systems could be further developed as molecular diagnostic tools [180], [181]. In a recent case, Collins and co-authors developed a flexible three-layer device using silicone elastomers and cellulose matrices, containing freeze-dried insets with genetic circuits. The circuits were configured to express the lacZ β-galactosidase operon, hydrolyzing a substrate that caused a colorimetric output when exposed to a target analyte [182]. The authors optimized the device materials and reaction kinetics to complete the colorimetric output in less than 60 min. Using the same principles, they built prototype devices with freeze-dried circuits for the detection of anhydrotetracycline, Ebola virus RNA, and small molecules [183]. Jung et al. developed a cell-free biosensor for water contamination via RNA output sensors activated by ligand induction [184]. Such a system consisted of highly processive RNA polymerase, allosteric protein transcription factors and synthetic DNA templates, which contained a fluorescence-activation RNA aptamer sequence. The binding of a target contaminant could trigger the release of allosteric transcription factors and allow the transcription of the synthetic DNA template. The resulting product, which contained a fluorescence activation RNA aptamer, could be detected via its florescence. The further development of various sensors for small molecules has been reviewed elsewhere [166], [180], [185].

## 5 Summary and perspectives

With continuous effort to improve the efficiency of the CFPS system, a new higher record of 4 mg/mL was achieved with an *E. coli* lysate-based batch CFPS system [59] and up to 6 mg/mL with continuous exchange configurations [186]. In the case of a complete reconstituted PURE system, the productivity of a continuous exchange setup reached around 30 % of the total protein contained in the optimized PURE system, comparable to the level of overexpressed protein in *E. coli* cells [60]. However, one would still wonder: Is the current productivity upper limit of the CFPS system catching up to the efficiency within a living cell? It would be challenging to directly answer this question due to the lack of accurate data on the overall performance of a cell. Nevertheless, we could get a close estimate. During the 20-minute doubling time of an active growing *E. coli* cell, an average ribosome would need to synthesize around 55,000 peptide bonds, which is at least 1–2 orders of magnitude higher than the current performance of CFPS systems [25], [187]. Taking into account the rate of synthesis, current CFPS systems are even less efficient, considering the highest protein synthesis rate at around 5000 peptide bonds per hour per ribosome [188]. Therefore, further efforts are still needed to improve overall efficiency. This would require a more efficient energy regeneration system and a homeostatic environment to maintain the efficiency of ribosomes [25]. A set of systematic studies have revealed the limiting factors for the overall yield of the corresponding CFPS system, including fast depletion of substrates (energy precursors, NTPs and certain amino acids) [22], degradation of DNA templates, transcribed mRNAs and target proteins [190] and accumulation of inhibitory biproducts, such as free inorganic phosphates, which cause the fast decay of ribosomes [189]. Furthermore, optimizations based on the fully reconstituted PURE system revealed certain transcription and translation factors would be benefit for improving the final yield [191], [192]. In addition, molecular crowding agents and chaperons also showed beneficial effects on the overall yields [53], [193]. Finally, recent proteomic analysis could give valuable information of limiting factors for extract-based CFPS systems, which might vary depending on the individual strains and preparation procedures [194], [195], [196]. As stated above, ribosome, as the core translational apparatus, plays a vital role in the overall performance of the CFPS system. Therefore, achieving ribosome biogenesis in cell-free environment not only reveals the fundamental principles but also has many potential applications in synthetic biology. Pioneering work from Jewett’s group in ribosome synthesis, namely integrated synthesis, assembly, and translation (iSAT), has shown the generation of functional 30 S, 50 S subunits with in vitro transcribed rRNAs [197]. Moreover, individual purified ribosomal proteins that form the 30 s and 50 s subunits could be assembled with native rRNAs into a functional ribosome [187], [198]. Furthermore, such iSAT process could be validated within a double emulsion templated vesicle [199]. However, despite the successful assembly of functional ribosomes with in vitro transcribed rRNA for 30 S subunits, post-translational modifications of 23 S rRNA might be crucial [200], which certainly need to be further investigated to generate full reconstituted ribosomes. Despite the rapid development and expansion of CFPS technique, it is still not a straight forward lab routine, which might require certain optimization work to establish the best suitable system for particular applications. Commercially available CFPS kits, together with a set of detailed protocols [42], [201], [202], [203], might be a good starting point.

The current fast development of different compartmentalization methods and materials for the formation of different cell membrane mimicries. The functional membranes are essential for a minimal cell, including selective permeability, responsive to different environmental signals, which are fulfilled by a large group of integral membrane proteins [204], [205]. Even before application in the field of synthetic biology, CFPS system was intensively tuned to express membrane proteins, which were difficult to overexpress in vivo, due to their cytotoxicity. Devoid of living cells, CFPS system possess unique advantages for membrane proteins and has successfully overexpressed a large number of membrane proteins for both functional and structural characterization, prepared with different hydrophobic reagents, such as detergent, lipids (i.e., liposomes, nanodiscs or other model lipid bilayers) [206], [207], [208]. The main effort in membrane protein expression using CFPS system shifts towards the optimization of co-translational hydrophobic environments, which is certainly target dependent [209]. In sum, such advantage of CFPS system on membrane protein expression will certainly be beneficial for minimal cell projects, as exemplified in our recent effort to reconstitute a reversible membrane switch direct on the supported lipid bilayers [90]. Furthermore, efforts to improve encapsulation efficiency are still needed, especially when a protein-rich and highly viscous solution is used [210], [211], [212], [213]. As noted above, the current CFPS system is still far less efficient than the cellular system. The encapsulation process would further challenge the performance of the CPFS system. Parameters and conditions optimized from bulk solutions would need to be curated and validated in the microcompartment, taking into account the molecular crowding effect and stochasticity therein [214], [215], [216]. Taken together, these factors would have a direct impact if we move towards an autonomous self-reproduction system, which would require the self-replication of genetic materials, the *de novo* synthesis of ribosomes, the minimal unit for protein production, phospholipids, and the minimal divisome [154], [217], [218]. In this regard, the research toward a minimal cell has just begun.

Despite the fact that sustainable and ecologically friendly biomaterials have a clear benefit in the product-driven sector of synthetic biology, they nonetheless cost more than the material obtained by conventional chemical refining techniques. Therefore, there is a continuous demand to reduce the overall cost, which is certainly critical for the application of CFPS systems. Although there have been ongoing efforts to reduce the price of CFPS systems, only a small number of high-value protein products have been successfully produced using this method so far. There is still some uncertainty about the issue. How far does the CFPS method go beyond prototyping in the realm of synthetic biology? The development of CFPS pathways for certain metabolites would also benefit from a quantitative model [219]. However, the one of the current barriers is the lack of standardized data that quantitatively explain the performance of the in vitro metabolic network. Another factor making standardization difficult is the wide variety of host strain backgrounds. Recent results from proteome analysis on multiple *E. coli* lysates may offer broad directions for a possible metabolic network [194], [195], [196]. In addition, the convergence of technological advancements in artificial intelligence will accelerate the process of constructing mathematical and computational models as a corollary. As previously stated, the CFPS system is rapidly growing into a valuable production tool for protein-based drugs and in vitro diagnostics. Real-time on-demand protein synthesis could be particularly beneficial in instances with limited resources, such as the current global SARS-Cov-2 outbreak.

Despite the hurdles that now exist, the rapid advancement in synthetic biology via CFPS has gained widespread interest from the scientific communities, which will surely result in a more diverse application and may soon be a game-changer in the area.

## CRediT authorship contribution statement

All authors contributed to the conceptualization, writing, reviewing and editing this manuscript.

## Declaration of Competing Interest

The authors declare that they have no known competing financial interests or personal relationships that could have appeared to influence the work reported in this paper.
